# A Review of the Effects of Cervical Cancer Standard Treatment on Immune Parameters in Peripheral Blood, Tumor Draining Lymph Nodes, and Local Tumor Microenvironment

**DOI:** 10.3390/jcm11092277

**Published:** 2022-04-19

**Authors:** Iske F. van Luijk, Sharissa M. Smith, Maria C. Marte Ojeda, Arlene L. Oei, Gemma G. Kenter, Ekaterina S. Jordanova

**Affiliations:** 1 Haaglanden Medical Center, Lijnbaan 32, 2512 VA The Hague, The Netherlands; 2 Center for Gynecologic Oncology, Amsterdam UMC, Meibergdreef 9, 1105 AZ Amsterdam, The Netherlands; m.c.marteojeda@student.vu.nl (M.C.M.O.); ggkenter@gmail.com (G.G.K.); e.jordanova@amsterdamumc.nl (E.S.J.); 3 Erasmus Medical Center, Doctor Molewaterplein 40, 3015 GD Rotterdam, The Netherlands; sharissasmith@gmail.com; 4 Laboratory for Experimental Oncology and Radiobiology, Department of Radiation Oncology, Amsterdam UMC, Location AMC, Meibergdreef 9, 1105 AZ Amsterdam, The Netherlands; a.l.oei@amsterdamumc.nl; 5 Department of Urology, The Netherlands Cancer Institute, Plesmanlaan 121, 1066 CX Amsterdam, The Netherlands

**Keywords:** cervical cancer, treatment, immune modulation, chemoradiation, neoadjuvant chemotherapy, tumor microenvironment, tumor draining lymph nodes, immunosuppression, immunotherapy

## Abstract

Cervical cancer remains a public health concern despite all the efforts to implement vaccination and screening programs. Conventional treatment for locally advanced cervical cancer consists of surgery, radiotherapy (with concurrent brachytherapy), combined with chemotherapy, or hyperthermia. The response rate to combination approaches involving immunomodulatory agents and conventional treatment modalities have been explored but remain dismal in patients with locally advanced disease. Studies exploring the immunological effects exerted by combination treatment modalities at the different levels of the immune system (peripheral blood (PB), tumor-draining lymph nodes (TDLN), and the local tumor microenvironment (TME)) are scarce. In this systemic review, we aim to define immunomodulatory and immunosuppressive effects induced by conventional treatment in cervical cancer patients to identify the optimal time point for immunotherapy administration. Radiotherapy (RT) and chemoradiation (CRT) induce an immunosuppressive state characterized by a long-lasting reduction in peripheral CD3, CD4, CD8 T cells and NK cells. At the TDLN level, CRT induced a reduction in Nrp1+Treg stability and number, naïve CD4 and CD8 T cell numbers, and an accompanying increase in IFNγ-producing CD4 helper T cells, CD8 T cells, and NK cells. Potentiation of the T-cell anti-tumor response was particularly observed in patients receiving low irradiation dosage. At the level of the TME, CRT induced a rebound effect characterized by a reduction of the T-cell anti-tumor response followed by stable radioresistant OX40 and FoxP3 Treg cell numbers. However, the effects induced by CRT were very heterogeneous across studies. Neoadjuvant chemotherapy (NACT) containing both paclitaxel and cisplatin induced a reduction in stromal FoxP3 Treg numbers and an increase in stromal and intratumoral CD8 T cells. Both CRT and NACT induced an increase in PD-L1 expression. Although there was no association between pre-treatment PD-L1 expression and treatment outcome, the data hint at an association with pro-inflammatory immune signatures, overall and disease-specific survival (OS, DSS). When considering NACT, we propose that posterior immunotherapy might further reduce immunosuppression and chemoresistance. This review points at differential effects induced by conventional treatment modalities at different immune compartments, thus, the compartmentalization of the immune responses as well as individual patient’s treatment plans should be carefully considered when designing immunotherapy treatment regimens.

## 1. Introduction

Despite screening and population-based vaccination programs, cervical cancer remains an important public health problem worldwide. As cervical cancer is the result of persistent infection with the human papilloma virus (HPV), it is considered an immunogenic tumor. Expression of the viral oncogenes E6 and E7, results in dysregulated cell cycle control and apoptosis [1].

Immune escape is considered a hallmark of cancer, as tumors evolve to evade immune recognition [2,3]. It is well known that paracrine, endocrine, and cell-cell interactions between the tumor cells and the tumor microenvironment (TME) influence disease development and prognosis, and the TME is considered an important prognostic factor. Conventional therapies can influence the microenvironment and anti-tumor immunity. At the same time, the TME will influence the effectiveness of therapy. There is growing awareness of the importance of a durable anticancer immune response induction and the key role it plays in achieving treatment success.

Traditionally, the treatment of cervical cancer consists of surgery, chemotherapy, and radiotherapy, or a combination of these modalities, depending on disease stage and patient characteristics. Those with stages Ib1 and Ib2, receive radiotherapy and concurrent chemotherapy (chemoradiation) or radical hysterectomy with pelvic lymphadenectomy. Patients with locally advanced cervical cancer, FIGO stage ranging from Ib3 to IVa, receive different treatment options, which are generally tailored according to international guidelines. These treatments include radiotherapy with or without concurrent chemotherapy, surgery for pelvic lymph nodes removal and consequent radiotherapy—this may involve consequent chemotherapy or not. In case of contraindications for cisplatin-based chemotherapy, radiotherapy can be combined with hyperthermia (thermoradiation). Treatment for the higher stages or recurrent disease is often individualized [4].

Primary (chemo) radiation is an effective treatment for locally advanced cervical cancer, with a five-year pelvic control rate of 87% and cancer-specific survival of 79% [5]. Patients with large tumors (>4 cm) or extension beyond the cervix to parametria or pelvic lymph nodes are less likely to be cured and have survival rates of 60–70% [6]. The standard treatment of choice is often external beam irradiation and brachytherapy in combination with cisplatin.

The local immune infiltrate of the tumor undergoes dynamic changes, where a shift takes place from a pre-existing immune response into a therapy-induced immune response. After primary treatment for cervical cancer, several changes in the immune infiltrate are described, that are highly associated with survival. These are the number and functional orientation of CD4+ and CD8+ T cells and the presence of M1 type macrophages [7,8,9,10].

Effective anti-tumor immunity can be achieved via the enhanced tumor associated antigen presentation of dendritic cells (DCs), the promotion of productive T cell responses, and by overcoming immunosuppressive signaling by malignant cells in the TME. Immunosuppression within the TME is usually a result of checkpoint molecule expression, of which cytotoxic T-lymphocyte associated protein 4 (CTLA-4) and programmed-death receptor (PD-1) are the best known. In the recent past, focus has shifted to exploring therapeutic possibilities by blocking these checkpoints. For several malignancies, such as melanoma and non small-cell lung cancer, long-lasting durable responses have been achieved with CTLA-4 inhibitors such as ipilimumab [11] and PD-1 inhibitors such as pembrolizumab [12,13,14]. After these successes and further investigation, FDA approval has now been given for immune checkpoint inhibitor use in multiple malignancies including lung cancer, bladder cancer, Hodgkin lymphoma, renal cell cancer, and head and neck cancers. In addition to the abovementioned conventional treatment options for cervical cancer, recently, much attention has been given to the addition of immune modulators for selected patient groups. Pembrolizumab was approved by the FDA for advanced cervical cancer in 2018 [15].

### Immunomodulatory Effects by Conventional Treatment Modalities

The direct cytotoxic effect of radiotherapy involves creating DNA damage that causes tumor cell killing, primarily via apoptosis. This leads to necrosis and mitotic catastrophe in the tumor cells. Delivery of localized radiation may lead to systemic responses at distant sites, a phenomenon known as the abscopal effect. This has been attributed to induction and enhancement of the endogenous anti-tumor innate- and adaptive immune response. It is important to realize, that there is a delicate balance between activation of the immune system and immunosuppression induced by radiotherapy. Radiotherapy causes radiation-induced inflammation and damages endothelial cells. Damaged vessels inhibit the infiltration of CD8+ T cells into the tumor, and immunosuppressive pathways are activated. Radio-resistant suppressor cells such as tumor-associated macrophages (TAM’s) with the M2 phenotype, myeloid derived suppressor cells (MDSC’s), and regulatory T cells (Tregs), accumulate in the tumor microenvironment [16].

By induction of immunogenic cell death (ICD), radiotherapy affects the immune response releasing new antigens to the components of the immune system [17]. Furthermore, radiotherapy leads to increased expression of specific surface molecules on the irradiated cells, which makes them more susceptible to cytotoxic T cell-mediated killing. Moreover, radiotherapy leads to the release of cytokines attracting T cells towards the irradiated tumor [18] and increases the release of damage-associated molecular patterns (DAMPs), which attract and activate cells of the innate- and adaptive immune system. The abovementioned events, can shift the balance toward an immunoreactive, as opposed to an immunosuppressive, microenvironment leading to the recruitment and activation of cytotoxic T cells [19,20]. The effects of brachytherapy (BT) on the immune response are assumed to be of lesser impact than external beam radiation therapy [21], however, a single dose of brachytherapy potentiates the anti-tumor immune response by increasing immune cell trafficking [22].

The impact and effect of chemotherapeutic agents on the functional and phenotypic characteristics of immune cells depends greatly on the type of drug and the treatment schedule. In general, chemotherapy modulates the tumor immune response via different mechanisms: 1. induction of immunogenic cell death which, similarly to radiotherapy, stimulates phagocytosis and DC maturation, and 2. induction of lymphodepletion and subversion of immunosuppressive immune responses. High-dose chemotherapy results in myelodepletion and lymphodepletion, while low-dose treatment has more anti-angiogenic and immunomodulatory effects [23,24].

Cisplatin and carboplatin are traditionally and currently the most applied drugs in cervical cancer treatment. The induction of cancer cell apoptosis, as a result of their covalent binding to DNA, is believed to be their main mechanism of action. Part of the anti-tumor effects of these platinum compounds, occurs through modulation of the immune system. The four main mechanisms of immune modulation by cisplatin are the following: 1. upregulation of human leukocyte antigens (HLA)-I, 2. recruitment and proliferation of effector cells, 3. upregulation of the lytic activity of cytotoxic effectors by activation of the caspase cascade, and 4. downregulation of the immunosuppressive environment [25,26,27,28].

Apart from these stimulating effects, chemotherapy and radiotherapy are also associated with negative effects on the immune state. Certain cells of the immune system, e.g., lymphocytes, can be rapidly dividing which makes them vulnerable to radiation and chemotherapy [29]. Generally, lymphocyte counts often decrease through the course of pelvic radiation [30]. Radiotherapy induces apoptosis in mature natural killer cells (NK cells) as well as T and B lymphocytes [17].

In cervical cancer patients, those with a rapid response to chemoradiation are more likely to remain disease-free for longer periods, but the underlying mechanisms for differences in response to treatment are not well understood. The effects of radiation per se on cervical cancer tumor infiltrate and immune cell composition, such as shifts in regulatory T cells and cytotoxic effector cells, have been studied recently in varying patient groups and in different research settings. In this review, we describe in detail what is known about the immunological effects of radiation therapy (RT), chemoradiation (CRT), and neoadjuvant chemotherapy (NACT) at three different levels of the immune system; effects on 1. peripheral blood (PB), 2. tumor-draining lymph nodes (TDLNs), and 3. the tumor microenvironment (TME). There is an urgent need to further explore the immune context of this immunogenic tumor, in order to determine the best timepoint to add immune checkpoint inhibitors (ICI’s) during traditional treatment regimens.

## 2. Methods

A systematic review was intended for this search. An extensive literature search was performed using the PubMed and Embase databases. The search strategy was built in cooperation with the medical librarian and resided upon the following cornerstones: cervical carcinoma, radiotherapy, chemoradiotherapy, (neo adjuvant) chemotherapy, the TME, tumor draining lymph nodes, and the immune system. A multitude of synonyms was used to identify all potentially relevant articles, and judgement of relevance was performed by three reviewers (IvL, SS and MCMO). The conduction of measurements performed before and after treatment was an important factor during selection, as it allowed for description of alterations specifically during treatment (*n* = 24). All studies concerning the influence of the abovementioned therapies on the immune system and the immune cell composition of the TME in patients with cervical carcinoma were included. Publications in languages other than English were excluded. The references of all fully read articles were checked for potential relevant records. As this appeared to yield more information regardless of the complicated and extensive search, in our opinion, it is more accurate to deem this review a narrative review.

## 3. Results

Immunological effects of chemotherapy and radiotherapy in patients with cervical cancer, can be divided into three main groups based on the effects on immune cells in: 1. peripheral blood (PB), 2. tumor-draining lymph nodes (TDLNs), and 3. the tumor microenvironment (TME). Different types of (chemo)radiation for different stages of cervical cancer are used and may vary in intensity, field, and type of radiotherapy. The most commonly applied form of therapy for locally advanced disease, is external beam radiation (EBRT) with or without internal brachytherapy (BT), usually in combination with cisplatin-containing chemotherapy. Summary of the content and characteristics of the relevant studies describing the abovementioned changes are displayed in Table 1, Table 2 and Table 3, respectively.

### 3.1. Effects of Radiotherapy and Chemoradiation on Immune Cells in Peripheral Blood

The following articles describe the effects of traditional treatment modalities on peripheral blood immune cells (Table 1).

Yamazaki et al. studied the effects of radiotherapy on the activity of NK cells, on the assumption that brachytherapy may have less influence than EBRT [21]. The overall number of lymphocytes and NK activity was significantly reduced after EBRT with or without brachytherapy. Only brachytherapy, however, produced little change in NK activity.

Lissoni et al. studied the effects of RT (EBRT) on lymphocytes, with or without concurrent chemotherapy (CRT) [31]. A decrease in total lymphocyte numbers of 50% or more was observed in 90% of patients. The minimum values of NK and cytotoxic T cells (CD8+) occurred after 2–3 weeks of radiotherapy, after which there was no further drop.

Bachtiary et al. focused on changes in lymphocyte subsets in cervical cancer patients with stage Ib-IVa receiving EBRT and patients treated with CRT [32]. All T cell subsets and B cells were significantly reduced in both treatment groups directly after treatment. At 24 weeks after treatment only B cells had recovered to pre-treatment levels. In the only RT group Tregs and NK cells had reached baseline values. In the CRT group, these were still significantly reduced. The CD4+/CD8+ ratio was still significantly reduced in both treatment groups. The mean lymphocyte number at the end of the treatment was lower in patients with stable disease (SD) or progressive disease (PD) compared to those that responded to treatment (completely or partially).

The aim of Eric et al. was to determine the absolute number and percentage of the specific PB lymphocyte subpopulations [33]. In all patients the immune cell subset absolute numbers declined. After RT the percentage of CD8+GrB+ lymphocytes was significantly higher compared to patients before RT, while the percentage of CD56+ and CD56+NKG2D+ lymphocytes was significantly lower only before RT compared to healthy volunteers.

Van Meir et al. described the impact of standard CRT on different aspects of the immune system in patients with stages Ib-IVa [34]. Within 48 h after the start of treatment, a decreased absolute number of circulating lymphocytes was found. This decline in lymphocytes lasted until 6 weeks after completion of therapy. During treatment with CRT, the frequency of both CD4+ and CD8+ T cells dropped, and CD4+ cells displayed a 2.7 fold increased expression of PD-1. In vitro blocking of PD-1 with nivolumab increased T cell reactivity in all five samples taken before the RT but was less successful in restoring reactivity after RT had started. An increase in both circulating monocytes and myeloid-derived suppressor cells (MDSC’s) and an impaired capacity of antigen-presenting cells (APC’s) to stimulate allogenic T cells was found after CRT.

Wang et al. analyzed the PBMCs of patients with stage Ib2-IIB that underwent surgery and different NACT regimes: TP (cisplatin+paclitaxel), TC (carboplatin), and TN (nedaplatin), before and after treatment. Two groups labelled as observation and control group were made, with the observation group receiving NACT and the control group surgery alone. Although there was not a significant difference in most of the parameters between both groups before and after treatment, CD4+/CD8+ and NLR were significantly decreased after treatment in both groups [35].

Rui et al. assessed the effects of RT with concurrent CRT on the local and systemic immune compartments in patients with stage IIa-IVa. The CD3+CD4+ T cell frequency was significantly lower in the mid-treatment and after-treatment samples, compared to the pre-treatment samples. The CD3+CD8+ T cell frequency significantly decreased three weeks after starting the treatment and slightly increased at the end of the treatment. Conversely, the mid-treatment and post-treatment Treg cells significantly increased mid-treatment and at the end of the treatment. Furthermore, the PD-1 expression on CD4+ T cell subsets and the CD8/Treg ratio significantly decreased after CRT. The authors also assessed the effects on TCR diversity with RNA sequencing and whether it could have an effect on prognosis. In this respect, patients presenting high TCR unique clonality before CRT had significantly higher PFS and OS, compared to recurrent patients. However, overall TCR diversity significantly decreased after CRT [36].

In conclusion, both EBRT and CRT lead to a reduction of all lymphocyte subsets immediately upon onset of therapy. The creation of this immunosuppressive state seems to occur regardless of chemotherapy administration. For NK cells, the deepest drop was noted mid-treatment; for all T cell subsets this decline continued until the end of treatment, lasting up to 6 months post-treatment. Moreover, the CD4+/CD8+ ratio and NLR were decreased after treatment indicating a suppressed hematological immune state. The patients that tend to better recovery of the immune subsets seem to be better responders [32]. Nevertheless, effects on peripheral blood seem to reflect a state of immunosuppression in cervical cancer patients after undergoing treatment.

### 3.2. Effects of (Chemo) Radiation or Chemotherapy on Immune Cells in TDLNs

Three articles described the effects of different treatment modalities on the tumor draining lymph nodes (Table 2). In these studies, all TDLNs were removed at surgery.

Fattorossi et al. made a comparison between the changes in lymphocyte subsets in TDLNs from patients with no prior treatment, prior (platinum containing) chemotherapy, and prior treatment with chemoradiation [37]. In addition to what is noted in Table 2, in all TDLNs the frequency and suppressive activity of regulatory T cells remained unchanged. The changes in the TDLNs were only partly mirrored in PB, demonstrating that the immune modulation is less outspoken in PB than in TDLNs. TDLNs of patients who achieved complete/microscopic response contained significantly higher proportions of activated CD4+ cells and NK cells, and significantly reduced proportions of Tregs.

Battaglia et al. published two studies on immunological changes in TDLNs in patients treated with chemoradiation. In the first study, the phenotype and function of CD4+ cells expressing the semaphorin III receptor neuropilin-1 (Nrp1), a Treg marker, was investigated in TDLNs and PB [38]. In TDLNs, Nrp1 expressing Tregs showed higher levels of FoxP3 and various other markers of activated Tregs such as CD45RO, HLA-DR, and glucocorticoid-induced tumor necrosis factor (GITR). In PB, these changes were not found. Therefore, function of Nrp1+Tregs seems to depend on the anatomical location. In TDLNs of patients after CRT, Tregs and Nrp1+Tregs dropped in relation to reduction of tumor mass.

In the second study [39], the immune state was compared between non-irradiated TDLNs (NI-TDLNs) of patients undergoing chemotherapy, TDLNs of patients undergoing low-dose chemoradiotherapy (LD-TDLNs), and TDLNs obtained from patients receiving high-dose chemoradiotherapy (HD-TDLNs) [39]. In LD-TDLNs, an enhanced anti-tumor response was found compared to NI-TDLNs. HD-TDLNs had an unfavorable tolerogenic/immunogenic dendritic cell ratio and a higher level of CCR4 expressing Tregs. The Th1 and Tc1 polarization found in LD-TDLNs was lost.

Altogether, CRT seems to improve the anti-tumor immune response in the TDLN by promoting activation of helper CD4+ and cytotoxic CD8+ T lymphocytes, and downregulating Tregs. The type of therapy does not differ in its effect on the drop in Tregs. The patients with a pathological response after surgery display more activated immune cells in their TDLNs. Low dose CRT seems the best regimen for immune activation and high dose led to the most impaired immune status.

### 3.3. Effects of Radiotherapy and Chemoradiation on Tumor Cells and the Tumor Microenvironment (TME)

In order to map out the effects of RT and CRT on the TME, a total of seven articles describing the local immune infiltrate before and after therapy were selected. Detecting immunological changes at the level of the TME during therapy requires sequential sampling of irradiated tissue. Disease stage, therapy regime, type of cells, and time points are specified in Table 3.

Qinfeng et al. reported on samples taken at different time points during the course of RT (Table 3) [40]. Due to tumor necrosis, it became exceedingly difficult to obtain representative biopsies after 20 Gy and 30 Gy. CD8+ T cell levels dropped significantly after 10 Gy, but not further after 20 or 30 Gy. Furthermore, in the biopsies taken after 30 Gy, OX40 T cells were significantly increased. The decrease in cytotoxic T cells and the relatively stable status of FoxP3+ Tregs indicated the relative radioresistance of OX40+ and FoxP3+Tregs.

In the study by Dorta-Estremera et al., TILs were analyzed over the course of CRT [41]. A significant decline in total T cells, Tregs, CD4+ T cells, and CD8+ T cells was observed in the first week of treatment, followed by variable expansion at weeks 3 and 5, that surpassed the levels at baseline. In addition, expression of CD69, which is a marker for activation, increased significantly over time in CD4+ and CD8+ cells. The highest proportion of CD86 expression on CD11c+CD11b- myeloid cells occurred at week 1 and week 5. Notably, the majority of the changes noticed at the level of the tumor were not observed in PB samples collected at the same time points, indicating the importance of sampling different tissues and locations for understanding specific therapy effects.

Berenguer Frances et al. performed a prospective study where changes in the TME were studied before initial treatment with CRT and brachytherapy with high-dose-rate brachytherapy (HDR) or pulsed-dose-rate brachytherapy (PDR) [42]. Macrophage rates, reflected by CD68 for pan macrophages and CD163 for M2 macrophages, were decreased, while there was a significant increase in PD-L1 expression after HDR brachytherapy.

Tsuchiya et al. described alterations in the expression of various proteins related to tumor immunity as well as tumor infiltrating CD8+ cells and FoxP3+ T cells induced by RT and CRT [43]. No differences in the expression of these proteins were found between patients with a pathological complete response (pCR) and those without a pathological complete response (non pCR). PD-L1 on tumor cells showed a significant increase after CRT. CD8+ T cell and Treg infiltration decreased significantly after CRT. Patients with higher CD8+ T cells or FoxP3+ T cells and PD-L1 expression on tumor cells in the pre-treatment specimen had higher OS. Moreover, patients PD-L1+ tumors had a significantly lower out-of-field recurrence rate.

Cosper et al. aimed to acquire insight into the evolution of treatment response and mechanism of resistance in patients receiving CRT [44]. Paired analysis with immunohistochemistry could be performed on 20 patients (out of 115). With a median follow up of 3.1 years, 13 patients had no evidence of disease (NED) and seven patients were dead of disease (DOD). Tumors from NED patients showed a harmonized response to therapy with strong enrichment in pathways related to the immune system in pre- and mid-treatment specimens, whereas DOD patients had 36-fold less altered genes during CRT. Tumor associated lymphocytes decreased in all patients, but DOD patients had a much greater decrease compared to patients with no evidence of disease (73% vs. 37% decrease, respectively). In tumors of patients with NED, higher expression of genes upregulated upon CD8+ T cell activation were found in the mid-treatment biopsy, including granzyme A, perforin, and granulysin. In samples from DOD patients, more HPV E6/E7 gene expression was retained during chemoradiation. In terms of exhaustion markers, DOD patients had decreased CTLA-4 and PD-1 expression, compared to the NED group. CRT decreased CD8 T cells among all patients, however, DOD patients showed significantly less infiltration in the mid-treatment biopsies compared to NED patients.

Lippens et al. studied patients receiving CRT prior to surgery [45]. Pre- and post-treatment specimens were stained for T cells, B cells, macrophages, PD-L1, and IL33. Depending on the number of positive cells, patients were divided into a low score or high score group. A total of 13 patients showed a pathological complete response (pCR). More pCR was seen in patients where pretreatment biopsies showed CD8 = CD3, CD8 ≥ CD4, positive IL33 tumor cell scores, and IL33 in immune cells < tumor cells. DSS was better in patients with high CD8 scores in the pretreatment biopsies, patients with CD8 ≥ CD4, CD163 > CD68, or PD-L1 > 5%. Patients with decreasing CD8 and CD163 scores during treatment showed more pCR, and those with increasing CD8 or decreasing IL33 scores showed a worse DSS. During follow-up, more metastases were seen in patients with an increasing CD3 score or unchanged or increased PD-L1 immune cell score.

Rui et al. analyzed primary tumor biopsies and PBMCs throughout patient treatment with CCRT [36]. The TME showed a decrease in tumor cell PD-L1 expression after 3 weeks, which further decreased after CCRT compared to pre-CCRT. The same was seen in the tumor-infiltrating CD4+ and CD8+ T cells.

When comparing TIL infiltrates in the TME before, during, and after treatment, it can be stated that the TME is dynamic and that after the primary decrease of the lymphocyte subsets the TME can gradually develop into a more immunogenic environment with expansion of several of these subsets. CD3+, CD4+, and CD8+ cells decline at first and in some cases this continues during the whole course of CRT, but expansion is also described after 3–5 weeks of treatment [41]. Immunosuppressive T cell subsets remained stable in several studies [40,41]. Therefore, these cells seem more radioresistant. Moreover, PD-L1 and CTLA4 expression were described as stable during the course of CRT. On tumor cells, PD-L1 expression was even higher which correlated with better OS [43]. High CD8+ infiltrate or high PD-L1 expression after treatment correlated with more metastasis [45], which is in contradiction with other studies [43].

The six studies described below, were not explicitly the result of the literature search, as they only describe the pre-treatment analysis of tissue in relation to treatment outcomes. For the purpose of later comparison and relevance to patient outcomes, we decided to add the following studies (Table 4).

Enwere et al. assessed expression of PD-L1 and intratumoral CD8+ T cell density in biopsies before CRT [46]. There was no correlation between CD8+ or PDL1 expression in regard to PFS or OS after CRT.

The study performed by Rocha et al. assessed biopsies before CRT [47]. A comparison of the expression of exhaustion markers, tumor-associated macrophages, and lymphocytes was made by immunohistochemistry between responders and non-responders. PD-1, PD-L1, and PD-L2 expression was higher in the stromal immune cells compared to peritumoral immune cells. Responders showed high numbers of CD8+ T cells in the stromal and peri-tumoral region, compared to non-responders.

Someya et al. performed two retrospective studies exploring the correlation between the number of TILs before treatment (with either RT or CRT) and outcome [48,49]. Significantly higher DSS was seen in patients with high numbers of CD8+ and FoxP3+ T cells before the start of treatment. Pre-treatment CD8-counts significantly predicted treatment outcomes in patients receiving low irradiation doses (group with an EQD2 of HR-CTV D90 of <70%).

The same author performed a retrospective study [49] in which 70% of the cases from the previous study, comparing pre-treatment TME characteristics to treatment outcomes, were included. This study categorized patients’ TME based on the T cell subtypes: inflamed, excluded, and desert. Cold tumors had significantly worse DSS. Furthermore, patients with high to intermediate CD8+ T cell counts before treatment showed significantly better prognosis. Moreover, low FoxP3+ counts and low HLA-I expression were associated with worse prognosis. Hot tumors, associated with better prognosis, had higher expression in PD-L1 as well as HLA-I.

The study performed by Komdeur et al. determining CD103+ as a possible predictive biomarker for cervical cancer, made a distinction between intraepithelial and stromal tumor-infiltrating CD8+ lymphocytes, pointing out that CD8+ CTLs expressing CD103+ were more prominent in the epithelial region and might have a higher effect on prognosis [50]. Patients in the CRT group showed reduced CD103+ infiltration compared to those receiving surgery alone. The CRT group showed significantly increased OS and PFS with higher CD103+ infiltration. This study also analyzed cervical cancer data from The Cancer Genome Atlas (TCGA) and correlated CD103 mRNA expression with prognosis and treatment regimen (radiotherapy vs. surgery). In this respect, patients with high CD103 expression showed significantly higher DSS. Furthermore, a strong association between CD103 expression and expression of exhaustion markers such as CTLA-4, PD-1, and PD-L1 was observed.

Kol et al. analyzed pre-treatment specimens before surgery alone, RT, or CRT in order to find the prognostic value of CD103+ and Stimulator of Interferon Genes (STING) protein expression levels and treatment outcome [51]. Similarly to Komdeur et al., TCGA data was analyzed and CD103 and STING mRNA expression was correlated with prognosis and treatment regimen. In both patient groups, those with high STING protein level expression and CD103+ expression demonstrated better outcomes in terms of DSS and DFS.

In conclusion, PD-L1 expression does not seem to be a clear prognostic factor. Higher rates of CD8+ T cells, CD103+ cells, STING, and, in general, hot tumors were all associated with improved patient survival and therapy response.

### 3.4. The Effects of Neoadjuvant Chemotherapy (NACT) on the TME

Patients with lower stages of disease sometimes receive NACT in order to reduce the tumor load before surgery. This is currently a topic of discussion as it is debated if this approach is superior to standard CRT regimes or surgery alone for the treatment of locally advanced cervical cancer [52].

A total of seven articles describing the local immune infiltrate before and after therapy were selected to map out the effects on the TME induced by NACT. Disease stage, therapy regime, type of cells, and time points are specified in Table 5.

Liang et al. analyzed pre-treatment biopsies of cervical cancer patients to determine the median CD8+ and FoxP3+ T cell infiltrate before NACT [53]. High density in CD8+ T cells in pre-treatment specimens was negatively correlated with lymph node metastasis. CD8+ T cell infiltration did not change after treatment, but the peritumoral and intratumoral FoxP3+ infiltrate was significantly lower in post-treatment biopsies. In addition, patients with pCR showed significantly lower FoxP3+ T cells in both areas after treatment when compared to non-pCRs. Low pre-treatment peritumoral FoxP3+ T cell rates were found to independently predict chemotherapy clinical effectiveness. On the other hand, a high ratio of intratumoral CD8+ T cells/stromal FoxP3+ T cells was an independent prognostic factor for both PFS and OS.

Palaia et al. collected tumor biopsies and blood samples from cervical cancer patients before NACT treatment [54]. Patients that responded to NACT underwent surgery. Pathological response to NACT was classified as pCR, pPR, or SD. Significantly improved response was observed in patients with high percentage of TILs and low NLR. Furthermore, patients with high numbers of stromal TILs and low NLR and PLR showed a significantly better response to NACT. There was no relation between high TILs at baseline and clinicopathological features. Moreover, there was no correlation between PD-L1 expression and treatment response. A significant positive correlation was found between TIL percentage and PD-L1 expression on tumor cells.

Meng et al. studied the expression of CD8, PD-1, and PD-L1 in cervical cancer patients with a history of NACT. Additionally, 30 patients presenting benign disease of the uterus were recruited as a control group [55]. PD-L1 and PD-1 expression were found in 70% and 68% of the cases, respectively. Most PD-1+ T cells and CD8+ T cells were found in the periphery rather than intratumorally. Additionally, higher FIGO stage, lymph node metastasis, and lymphovascular space invasion (LVSI) were associated with higher PD-L1 expression.

Recently, our group also assessed the TME composition before and after NACT [56]. The immunomodulatory effects were only present in the stroma and changes were only significant in the chemotherapy combination therapy group (cisplatin and paclitaxel). A significant increase in the cytotoxic CD8+ T cell, FoxP3+CD8+ T cell, and Tbet+CD8+ T cell infiltrates was observed. Furthermore, patients receiving the combination cisplatin and paclitaxel also experienced a decrease in proliferating CD4+ cells (CD3+CD8−Ki67+) and proliferating conventional Tregs (CD3+CD8−FoxP3+Ki67+) after treatment.

Another study performed by Liang et al. assessed the expression of PD-1, PD-L1, CD3, CD8, and CD4 on TILs before and after NACT [57]. After NACT, PD-L1 expression increased and was observed in 95% of the patients (>1%). Interestingly, a significant decrease in PD-L1 expression was observed in patients that responded to chemotherapy. This study also found an increased DFS in patients with decreased PD-L1 expression. Nevertheless, a significant association between infiltration of CD8+ TILs and PD-L1 expression was observed, and increased CD8+ TILs after treatment were linked to higher DFS.

Zhang et al. assessed the immune infiltrate in patients that received chemotherapy only or in combination with surgery. In this study 43 patients (out of 60) provided matched pre- and post-NACT samples [58]. Staining scores revealed that baseline T cells, B cells, helper T cells, cytotoxic T cells, PD-1+, macrophages, and NK cells were the major representative immune cells and that there was a significant, direct correlation between PD-L1 expression on tumor cells and TIL rates. Immune infiltrate profiling revealed four different clusters of patients according to immune cell densities: cluster 1 (immune-active), cluster 2 (immune-medial), cluster 3 (immune-deficient), and cluster 4 (immuno-NK). Of these clusters, the immune-deficient had the lowest PFS. Immune-NK had higher PFS compared to the immune-medial cluster. The immune-active cluster showed the longest PFS. After NACT, mostly with cisplatin, there was a significant decrease in Ki67 and PD-L1 expression specifically on tumor cells, whereas a significant increase in CD4+, CD8+, CD20+, CD56+, and PD-1+ cells was observed in >50% of the patients. Moreover, a depletion in CD14+ myeloid cells was found. These changes were significant in responders compared to non-responders. Interestingly, unsupervised cluster analysis showed that all clusters presented an increase in CD56+ NK cell infiltrate. Furthermore, the immune-deficient cluster showed the most prominent increase in TILs.

D’Alessandris et al. analyzed tumor samples before NACT and after surgery [59]. The CD8+ T cell infiltrate was more prominent than the CD4+ T cell infiltrate. Although less prominent than the lymphocytes populations, NK cell infiltrate was observed in 76% of the cases and macrophages in 95% of the cases. There were also significantly higher numbers of CD3+, CD4+, and CD8+ T cells when the PD-L1 score was >10%. PD-L1 positivity on tumor cells was associated with high TIL rates and increased PD-L1 expression on inflammatory cells. Patients showing complete response presented with a high percentage of stromal T cells compared to those with progressive disease.

After assessing the effect of NACT in the TME, it can be concluded that chemotherapy provokes several important changes in cervical cancer. Responding patients tend to have higher TIL rates than non-responders [59]. After NACT, high intratumoral CD8+ T cells versus low peritumoral FoxP3+ cells is predictive for overall and progression free survival [53]. FoxP3+ T cells were significantly decreased after NACT with a significantly lower density in complete responders [53]. PD-L1 expression both on inflammatory and tumor cells had no relation to response [54,59]. Although expression of PD-L1 increased upon NACT, specifically in responders, PD-L1 expression was decreased [57]. Patients receiving NACT containing a combination with paclitaxel, tended to have stronger therapy induced T cell modulation with decreased CD4+ and Treg infiltrate and in that manner more potential to induce immunogenic cell death [56]. In responders, depletion of myeloid cells was also observed [58]. The majority of studies report that the most noticeable alterations in TILs occur in the stromal compartment.

## 4. Discussion

The aim of this review was to map out changes at the different levels of the immune system when traditional therapy in the form of chemoradiation or neoadjuvant chemotherapy is administered to treat cervical cancer. Elucidating these changes will pave the way to design rational trials incorporating immunotherapy, which can be administered at the most appropriate time point during multimodal treatment.

For cervical cancer, it proved difficult to determine the specific effect of either chemotherapy or radiation on immune parameters, as both treatments are often administered together in both different schedules and different regimens for locally advanced disease. Apart from clinical stage, other known factors such as HPV status and age may influence the individual immune response. This information was not specified in detail in the incorporated studies so these effects are not taken into account. Data on direct effects of chemotherapy on the tumor microenvironment are scarce. In the neoadjuvant setting, cisplatin-based chemotherapy is currently the treatment of choice [60]. Moreover, studies describing immunological changes following radiation therapy are scarce and heterogeneous. The difficulty to study and describe the exact changes at the tumor level lies in the fact that radiation often damages the tissue so thoroughly that the TME cannot be accurately studied. Therefore, immunological parameters at other sites, usually in peripheral blood or TDLNs, are studied as surrogates [32]. An important finding when taking into account the results of this review is that immunological changes at these different levels appear not to develop at the same pace and intensity.

### 4.1. Effects of Chemoradiation on Peripheral Blood

Pelvic RT, with or without CT, induces profound, unfavorable immunological changes in peripheral blood. Hematologic toxicity is frequently noted as 40% of active bone marrow is situated in the pelvic region. Already, within 48 eight hours after the start of treatment, a drop in lymphocyte subpopulations is apparent [34]. Lymphopenia is generally associated with chemoradiation [26,61,62]. NK cells diminish after treatment with standard therapy and are highly radiosensitive [63]. The deepest drop was found mid treatment. NK activity was found to be affected by many factors related to the treatment, such as the RT field, and patient characteristics. Because NK status can change quickly, it is deemed non-specific and difficult to apply as a prognostic marker. Transient suppression does not necessarily correlate with poor prognosis; some studies suggest that radiotherapy alone has a shorter-term suppressive effect on NK cells compared to when concurrent chemotherapy is administered [31,32]. NK and CD8+ cytotoxic lymphocytes may express the NKG2D transmembrane stimulatory receptor, which upon activation stimulates cytolysis and cytokine production by NK cells and provides costimulatory signaling for CD8+ activation. No significant increase in NK cells expressing this receptor was found [33]. Previous studies have however associated activated NK cells with adverse treatment outcomes in cervical cancer patients [64], therefore, this could be deemed beneficial.

CRT and RT were also associated with a prolonged T lymphocyte decline in all subsets which translated to adverse treatment outcomes [31,32]. Moreover, increased percentage of MDSC’s and monocytes, and upregulated PD-1 expression on circulating CD4+ T cells was observed [34]. Importantly, chemoradiation was able to induce a long-term reduction in suppressor T lymphocytes. The addition of chemotherapy to patients’ treatment regimens adds to hematological toxicity; after addition of chemotherapy, NK and Tregs returned to pretreatment levels only in the RT group [32].

Different combinations of chemotherapeutic agents induce different effects on the peripheral blood immune composition and in the local tumor immune microenvironment [35,56]. Most studies monitored patients receiving CT containing cisplatin alone. This treatment regime, administered with radiotherapy, induced a decrease in CD4+ and CD8+ T lymphocytes and an increase in immunosuppressive regulatory T cells and myeloid subsets [34,36]. However, CRT containing both paclitaxel and cisplatin seems to have a positive effect on anti-tumor immunity by increasing the CD4+/CD8+ ratio and NLR ratio in the peripheral blood [35]. Paclitaxel was found to promote MDSCs differentiation into mature DCs [65], therefore, the addition of paclitaxel to treatment regimens could be significant for modulating immunosuppressive cell populations thereby shifting the balance to a more immunoreactive environment.

Activation and proliferation markers were upregulated on CD8+ and CD4+ T cells after three weeks of CRT. Interestingly, DC activation was observed after one week in the same study [41]. Immunogenic cell death induced by chemotherapy and radiotherapy is known to release danger signals that in turn license DCs for CTL activation via cross-presentation [66], which could explain the abovementioned result.

The effect on PD-1 and PD-L1 expression in PB in the different studies is contradictory when administering chemoradiation. Some patients will experience an increase while others will experience a decrease [34,36]. Hereby, we argue that the contradiction in PD-1 and PD-L1 expression results could be due to differences in time point measurements. In this respect, studies in lymphomas have found that PD-1 expression in the tumor is very dynamic as the immune response evolves during treatment with chemotherapy [67]. Apart from treatment, other factors including IFN-γ [68], oncogenic and hypoxic signaling pathways, and post-translational modifications, have been found to influence the dynamic temporal expression of PD-L1 [69]. Peripheral blood PD-L1 expression is also dynamic, and factors such as IFN-γ also influence PD-L1 expression in the peripheral infiltrating immune cells [70]. Due to the dynamic nature of the PD-1/PD-L1 axis expression, we suggest that future studies should measure other factors influencing PD-L1 expression. Furthermore, different cut off values for PD-L1 expression were used among studies, therefore, baseline measurements could have affected post-treatment expression assessment differently in different studies. Alternatively, a decrease in immune checkpoint expression could also be explained by a decrease in lymphocytes, considering that PD-1 and PD-L1 upregulation is associated with TCR signaling and rearrangement [71,72].

### 4.2. Effects of Chemoradiation on Tumor Draining Lymph Nodes

The pelvic TDLNs are often the first station where metastasis appear and are considered important immunological checkpoints in cervical cancer. At radical surgery, these nodes are removed for staging. Earlier studies in TDLNs in other cancers clearly point to the major role played by factors such as type, stage, and distance from the primary tumor site in governing the immune activation of TDLNs. Fattorossi et al. strongly suggested an enhanced capacity of the local immune system to attack tumor cells as there was a significant enrichment of activated and effector type T cells, and predominance of CD8+ cells prone to produce IFNγ, indicating an enhanced capacity to mediate the anti-tumor effect. NACT did not have a reducing effect on the Tregs in this study. It is also important to note that the changes observed in TDLNs were only partly seen in the PB [37].

Differences in radiation dose for cervical cancer demonstrate different immune reactions at the level of the TDLNs. In TDLNs there is often T cell tolerance rather than activation thereby preventing immune attack and facilitating local tumor progression. Lymphocytes travel continuously between the PB and tissue through the lymphatic system and the failure or success of production of an adequate immune response against cancer cells in TDLNs is crucial for protection against metastatic growth. Battaglia et al. demonstrated that low dose chemoradiation created the most immune competent environment with Th1 and Tc1 polarization reflecting expansion of cytotoxic T cells. We argue that (LD) CRT could help restore the T-cell mediated anti-tumor response and hamper immunosuppressive T-cell functions in the TDLN in order to maximize response to immune checkpoint blockade. CRT could have an indirect positive effect on the anti-tumor T-cell mediated immune response by downregulating immunosuppressive T cells in the TDLNs. Considering TDLNs, it has been proposed that removal of uninvolved nodes carries the risk of eliminating a potential source of immune competent cells. The abovementioned studies support this view.

### 4.3. Effects of Chemoradiation on the Tumor Microenvironment

Suppression of several lymphocyte subsets in the TME after CRT was noted in most of the studies after which expansion of CD3+, CD4+, and CD8+ T cells was seen. Patients with a high CD8+ score compared to CD3+ or CD4+ showed more pCR. Patients with a low or declining CD8+ score post treatment also had a higher probability of achieving pCR [45]. This is in line with earlier findings that showed that high pretreatment levels of CD8+ are associated with better survival. Interestingly, in several studies FoxP3 and OX40 T cell subsets remained stable during the course of CRT, deeming them more radio-resistant. In the context of squamous cervical cancer, Treg counts have been associated with clinical outcome and radio resistance. The suppressive functions of Tregs are associated with hampered CD8+ T cell anti-tumor functions and reduced radiotherapy-induced tumor cell death [73]. Furthermore, the localization of Tregs in the TME is important. Peritumoral Tregs could suppress immune cells via direct interaction, creating a barrier that prevents CD8+ T cell infiltration, relegating them to the vicinity of the tumor [74]. RT alone vs. CRT exerts different effects on Tregs and affects T cell populations at different compartments. In this regard, Tregs in the stromal compartment remain stable when administering RT alone, in contrast to CRT which induces a decline in peritumoral Tregs [40,41]. A concurrent drop in Tregs and CD8+ T cells was observed when the treatment of choice was combination therapy [41]. We argue that these findings could be explained by co-trafficking of Tregs and CD8+ T cells, which has been previously reported in other types of cancer [75].

PD-1 and PD-L1 are often upregulated on tumor cells and myeloid cells, allowing tumors to avoid immune surveillance. Increased expression levels of PD-L1 impair T cell mediated immune responses in various solid tumors including cervical cancer [76]. Levels of PD-1 and CTLA4 remained stable after CRT in the results summarized in this review. Dorta et al. described a significant increase of PD-L1 expression 2 weeks after completion of brachytherapy [42]. Tsuchiya et al. also state that PD-L1 on tumor cells was significantly increased after CRT [43]. Better OS was correlated with higher expression of PD-L1 on tumor cells [43]. PD-1 expression during treatment with CRT in patients that died of disease [44] is a paradox that could be explained by decreased viral antigen activation of T cells and decreased immunoreaction [77]. The study by Cosper et al. revealed that mid-treatment immune landscape evaluation is an important predictive parameter, where the reduction of all TILs during treatment was significantly more reduced in patients who DOD. Patients with NED at the end of treatment showed enrichment in pathways related to the immune response. This may provide a better prognostic value as differences were greater mid-treatment [44].

### 4.4. Effects of Neoadjuvant Chemotherapy on the Tumor Microenvironment

The tumor-associated stroma is known to play a role in cancer progression and metastasis. Our findings confirm that the most prominent changes occur in this compartment, in favor of TIL increase. Stromal cells can play an important role in hampering immunity by recruiting, for instance, Tregs and MDSC’s and by suppressing the proliferation of cytotoxic T cells and helper T cells [78]. We found that NACT exerted a greater negative effect on peritumoral Tregs when containing paclitaxel/cisplatin compared to cisplatin alone. Additionally, combination NACT therapy also induced a positive effect on peritumoral and intratumorally cytotoxic lymphocytes, including those expressing the Tbet transcription factor [55,56]. In triple-negative breast cancer, increased Tbet expression positively correlated with CD8 expression and longer survival, when administering adjuvant chemotherapy [79]. Therefore, switching the balance of the stromal immune response towards a cytotoxic anti-tumor response using a combination NACT regime may improve prognosis and overall survival. When dividing patients into subgroups, the immune-reactive group had the best prognosis. This supports the idea of enhancing the immune compartment with additional therapy.

PD-L1 expression after treatment with NACT is variable in cervical cancer. Increased PD-L1 and PD-1 expression after chemoradiation or NACT treatment was observed in several studies [43,44,54,55,57,58], which could be attributed to chemoresistance. Some studies reported a reduction in PD-L1 expression after treatment, which co-occurred with a decline in TILs [36]. When there was a decline in PD-L1, this was associated with better DFS [57]. PD-L1 expression has been found to correlate with TIL counts [54,59], therefore, this could be explained by the reduction in TILs after treatment. Interestingly, in patients with longer DFS the PD-L1 expression was decreased after NACT, as opposed to those patients with shorter PFS [57].

### 4.5. Prognostic Value of Local Immune Biomarkers for Conventional Treatment and Immunotherapy in Cervical Cancer Patients

The vast majority of studies demonstrate that the pre-treatment state of the TME greatly affects treatment outcome. Rocha et al. reported higher pre-treatment CD8+ TIL levels before treatment in responders, compared to non-responders. This study demonstrated a positive correlation between high CD68+ cells and PD-1 expression in the stroma of non-responders, in addition to a negative correlation between stromal PD-1 expression and CD8+/PD-L1+ TILs. High CD8+ T cells, CD8+ CD103+, and FoxP3+ infiltration, and high PD-L1, STING, and HLA-I expression before treatment, were associated with longer DSS and OS as well as lower metastasis rates and recurrence [43,47,48,50,51]. Therefore, these findings suggest that patients with inflamed tumors respond better to chemoradiation compared to non-inflamed tumors.

PD-L1, PD-L2, and PD-1 expression in the pre-treatment specimen was reported by several studies [36,42,43,46,47,59]. In order to prevent chemoresistance associated with PD-L1 expression, a specific group of patients could benefit from ICI administration before chemoradiation. This is especially applicable to hot tumors, which show more prominent pre-treatment PD-L1 expression. Additionally, hot tumors show increased stromal PD-1, PD-L1, and PD-L2 expression before treatment [80].

Previous studies on HPV+ head and neck cancer have associated improved ICI response in patients presenting with pre-treatment immunosuppression caused by the PD-1/PDL-1 axis and in inflamed tumors [81,82]. The advantage of using immunotherapy in treatment naïve patients has been reported in previous studies since cellular stress induced by treatment can increase immunosuppression [83].

More advanced disease stages, which are usually treated with CRT/RT, were shown to be associated with increased immunosuppression, reduced CD103+ CTL counts, and STING expression. Moreover, prognosis was worse in patients with more advanced disease compared to patients with less advanced disease stages receiving surgery alone. Increased CD103+ CTL counts were shown to correlate with increased PD-L1 and PD-1 expression [50,51]. Previous studies have associated potentiation of anti-tumor immune response after RT with the STING pathway, which increases the antigen-presentation capacity in APCs through the Type I interferon pathway [84]. We conclude that, when considering disease stage and tumor T-cell phenotype status, results indicate that a subset of patients with PD-1/PD-L1 positive tumors, inflamed phenotype, and/or less advanced disease stage could benefit from immunotherapy before traditional treatment.

Altogether, these studies suggest that pre-treatment expression of markers associated with the immune response could be deemed as prognostic markers for conventional treatment outcome. This pretreatment information could help us to tailor management toward primary treatment while per-treatment information could guide us towards personalized adjuvant treatment.

Furthermore, we cautiously state that the abovementioned markers together with pre-treatment PD-1 and PD-L1 expression status might be used as predictive biomarkers for the immunotherapy response.

### 4.6. Future Views on Addition of Immunotherapy in Treatment for Cervical Cancer

Traditional treatment with radiation therapy alone, or in combination with chemotherapy, has been shown to induce an important suppression of the lymphocyte subsets causing an immunosuppressive environment, more or less similar in the peripheral blood and the local tumor immune microenvironment. In the TDLNs, low-dose chemoradiation seems to ignite an activation of immune cells. Low irradiation dose has been found to modulate the TME through immune reprogramming by release of danger signals improving the anti-tumor immune response [85]. Low dose irradiation induces normalization of the vasculature resulting in, for instance, increased T cell infiltration into the tumor nest, effector function potentiation, and increased recognition of tumor cells. Local low dose irradiation also results in potentiation of the systemic anti-tumor immune response through tumor antigen release and immune cell priming, which results in the regression and control of distant metastasis. This mechanism is known as the so-called abscopal effect [86,87]. Someya et al. found a positive correlation between high CD8 counts, lower brachytherapy doses (group receiving EQD2 of HR-CTV D90 of <70%), and improved control of distant metastases. There was, however, no association in the group receiving higher irradiation doses (group receiving EQD2 of HR-CTV D90 of >70%). This study also found higher HLA-1 and CD8 expression in hot tumors. Additionally, Rodriguez-Ruiz et al. reported that a single dose of brachytherapy leads to an increase of anti-tumor immune response by reducing vasculature and improving the trafficking of immune cells to the tumor [22]. Thus, we argue that these patients may experience improved control of distant metastasis probably partly due to increased immunogenicity and immune reprogramming, induced by lower irradiation doses. Furthermore, high dose radiotherapy, given in a hypofractionated regime, has been shown to increase surface expression of immunogenic molecules on cancer cells [88]. Interestingly, Someya et al. found slightly increased DSS in immune-excluded tumors compared to hot tumors. Therefore, we argue that low irradiation doses given in fractions could increase the immunogenicity of excluded tumors.

A general decrease of T cells—and in the case of the TME, a decrease in infiltration —was observed, especially in non-responders. Results from peripheral blood and the TME indicate that treatment with traditional therapy could potentiate the function of immunosuppressive immune cell populations such as MDSCs and Tregs. Radiation therapy is a known inducer of MDSCs chemoattraction through CCL2 accumulation and Tregs expansion via the adenosine pathway. These immune cell subsets play a key role in the suppression of T effector cells functions and radio-resistance, and determine response to ICI [73,83,89,90] which could explain why a decrease in T cell reactivity and count occurred together with an increased presence of immunosuppressive cells. Furthermore, Tregs return to baseline levels after CRT and RT in the blood and the local tumor microenvironment. Depletion of Tregs before treatment with RT could therefore minimize radioresistance and improve anti-tumor T cell response. Previous studies in renal cancer have demonstrated that immunotherapy aimed at Treg depletion, such as anti-CTLA4 and anti-CD25, could enhance local anti-tumor response and induce an abscopal effect [91].

Results hint that the effects on the T-cell mediated anti-tumor response induced by radiotherapy and chemoradiation are different. In this respect, expansion of CD8+ T cells after CRT has been shown [41], and in one study this occurred at a later time point [36]. Delayed post-treatment infiltration of CD8+ T cells has been previously described in the context of hypo-fractionated irradiation, which might explain this finding [92].

Furthermore, the effects on peripheral blood NK lymphocyte counts further suggest a need for immunotherapy before conventional treatment, since RT alone induces a significant decrease in this cytotoxic lymphocyte subset. Studies in mouse models have found that NK cell-aimed immunotherapy with CpG and Herceptin induced better anti-tumor effect before radiation therapy [93]. Overlooking our review, the findings hint at the need for initiating studies with immunotherapy before RT/CRT treatment to rescue cytotoxic lymphocytes and deplete immunosuppressive cells.

Administration of PD-L1/PD-1 blockade in the presence of antigen-naïve CD8+ T cells could induce dysfunctional CTLs and consequent resistance to ICI [94,95]. Therefore, irradiation before ICI treatment could induce antigen-specific CD8+ T cells that can be rescued later on. One of the proposed mechanisms for this phenomenon is the induction of radiotherapy-mediated immunogenic cell death (ICD) [17], which increases antigen presentation by APCs, increased HLA-I on irradiated cells and NKG2D receptor ligands [17,96,97]. Induction of ICD has also been attributed to chemotherapeutic agents [98]. This phenomenon might explain increased cytotoxic marker expression and delayed increase in CD8+ T cells after (chemo)radiation, as well as the association between high pre-treatment CD8+ T cell counts and improved treatment response. Altogether, administration of PD-L1/PD-1 blockade after or in combination with conventional treatment could be deemed more beneficial to counteract treatment resistance mechanisms and rescue exhausted cytotoxic T lymphocytes. Radiotherapy and chemoradiation induced an increase in PD-1 expression in both the peripheral blood and TME [34,47]. Moreover, PD-1 expression after CRT could indicate a significant increase in expansion of tumor-reactive immune cells [99]. Ipilimumab treatment, targeting CTLA4, after CRT has been approved by the FDA for irresectable melanoma [100]. This treatment, has been shown to sustain PD-1 expression on lymphocytes induced after CRT in cervical cancer patients, hinting that administration after CRT could improve the potentiation of a tumor-reactive environment. Interestingly, this approach is tolerated and effective in cervical cancer patients experiencing PD-1 upregulation after CRT [96].

In conclusion, the timing of immunotherapy might vary depending on the antibody of choice [101]. Nevertheless, it can be concluded that CRT/RT could be used to increase immunogenicity and increase tumor-reactive lymphocytes before treatment with anti-PD-1 and anti-PD-L1 agents.

Chemotherapy before surgery has been shown to induce negative modulatory effects on stromal Tregs, which are associated with an adverse prognosis. Positive modulatory effects are seen on cytotoxic T cells dependent on T-bet expression, which is associated with a good prognosis. This was especially observed with chemotherapy containing both cisplatin and paclitaxel, highlighting the importance of addition of the latter to the combination treatment regime.

Combining chemotherapeutic agents with ipilimumab has previously been found to increase the negative effects on Tregs populations compared to chemotherapy alone, in melanoma patients [12]. Treg depletion has been associated with adverse treatment outcomes, such as autoimmunity [102]. Stromal Tregs suppress anti-tumor immunity by direct suppression and by halting lymphocytes infiltration. Therefore, NACT alone could serve as a mechanism to reduce stromal Tregs count before treatment with immunotherapy. Furthermore, from our results it is clear that NACT is associated with an increase in PD-L1 expression, which indicates that administration of anti-PD-L1 after NACT could be beneficial for preventing chemoresistance and add to the desired therapeutic target.

### 4.7. The Current Status of Immunotherapy Implementation for Cervical Cancer Patients

The ideal timeline of immunotherapy and RT administration remains to be determined. Although there are several ongoing clinical trials, most do not study the impact of immunotherapy and radiotherapy sequence [99].

FDA approved pembrolizumab (2018) for the treatment of PD-L1+ (>1% of cells) recurrent metastatic cervical cancer tumors after therapy with traditional treatment. However, the response remains dismal with an ORR of 12.5% [103].

Nivolumab (anti-PD-1), previously approved by the FDA in melanoma patients, is currently being used in the CheckMate-358 phase I/II trial for the treatment of recurrent metastatic cervical cancer [104,105]. A continuation of the CheckMate-358 study with the addition of ipilimumab in treatment naïve patients showed PFS in 53% of the patients after 12 months.

The effect of administering immunotherapy sequentially or concurrently with CRT treatment in cervical cancer patients is currently being explored in other clinical trials such as NRG GY017, NCT02635360, and NCT02921269 [99,104,105].

Altogether, the best timing for the several immunotherapeutic possibilities is not clear from currently available data. Patients that have not received traditional treatment could be deemed as ideal candidates for immunotherapy, however, results from ongoing clinical trials are needed to confirm this.

## 5. Conclusions

This review describes changes in immune expression patterns at the level of the peripheral blood, tumor draining lymph nodes, and in the tumor microenvironment before and after traditional treatment for cervical cancer.

According to available data, RT with or without CT induces an overall and long-lasting immunosuppressive state in the peripheral immune system, especially prominent in patients with worse treatment outcome. At the level of the TDLN, CRT truncates Treg stability and numbers and potentiates the helper and cytotoxic T cell response, especially in patients receiving low irradiation doses. It is important to realize that effects of treatment on the TME, or at the level of the TDLNs, are only partially reflected in the peripheral blood and studies focusing on comparing those effects are much needed.

The effects that are accomplished by CRT and RT together with brachytherapy on the different immune compartments are heterogeneous involving both immunomodulatory and immunosuppressive effects, which are highly dependent on a range of factors such as dose, sequence of administration, and disease stage. The latter should be carefully considered since increased immunosuppression associated with advanced disease stages might be further worsened by CRT and RT, thus affecting response to immunotherapy. However, overall, Tregs showed treatment resistance. We suggest that the planning of immunotherapy regimens for patients with scheduled CRT and RT should be designed in a personalized setting, taking into account the abovementioned factors.

In addition, the immune state before CRT and RT is crucial since pre-treatment pro-inflammatory immune signatures were associated with longer OS and DSS, thus hinting at potential prognostic biomarkers.

We argue that when NACT is administered before radical surgery, ICI administration after treatment would be most appropriate. This approach is beneficial for downregulation of immunosuppressive T cell populations associated with anti-tumor immune response dampening.

Considering the fact that there is a significant group of patients with high tumoral immune checkpoint expression before treatment, future clinical trials should be aimed at exploring the benefit of immune checkpoint administration before conventional treatment. We argue that this specific group of treatment-naïve patients might greatly benefit from immunotherapy.

This review suggests that future studies using standardized protocols and cut-off values are desperately needed in order to validate specific prognostic and predictive immune markers. This will enable the selection of patients that could benefit from immune therapy and help determine the optimal time point during standard therapy to administer immune checkpoint inhibitors.

## Figures and Tables

**Table 1:** Effects of radiation or chemoradiation of peripheral blood lymphocyte subsets.

Author	N of Patients with cx ca	FIGO Stage	Therapy	Markers	Time Point	Results
Yamazaki 2002	16 (16/27)	Ibi–IIIb	EBRT and or BT 50 Gy	NK cells	Before, after trt, 2–6 months	NK activity ↓ after EBRT
Lissoni 2005	29	I–IV	EBRT (+/−CT) 50.4 Gy	CD3, CD4, CD8, NK (CD16/CD56)	Before start, thereafter weekly	NK, CD3 max ↓ after 2–3 wks, CD3, CD4 cell ↓ continued >2 weeks after end of trt, CD3 ↓ at end of trt in pts with SD or PD
Bachtiary 2005	34	Ib–IVa	EBRT *n* = 14 RT-CHT *n* = 20 40–50 Gy	Total T cells, T helper cells, T suppressor cells, T cytotoxic cells, NK cells, B cells, CD4/CD8 ratio	Before trt, after trt, 6, 12, and 24 weeks after completion of trt	24 Wks after trt: B cells = pre trt levels, T cells, Th cells, cytotoxic T cells ↓ in both groups, Treg, NK cells ↑ to pre trt levels in RT group
Eric 2009	39	IIb–IVa	EBRT 46 Gy + brachy 35 Gy *n* = 39	CD8, CD8NKG2D, CD8/granzymeB, CD16, CD16NKG2D, CD56, CD56NKG2D	Before trt, after trt	% All subsets ↑ after RT, In pts with PD: CD8, CD8NKG2D, CD16 and CD56NKG2D all not ↑
Van Meir 2017	30	Ib–IVa	EBRT + chemo *n* = 23 RT *n* = 6 HPT/RT *n* = 1 46 Gy	Lymphocyte and myeloid subsets, expression of co-stimulatory molecules, T cell reactivity, APC function	Before trt, midway, 3, 6, and 9 weeks after EBRT	All leucocytes ↓ after trt, during trt: CD4 and CD8 ↓, PD-1↑ on CD4 after blocking of PD-1 before RT: T cell reactivity↑, not after start trt, monocytes, MDSC’s ↑, stimulatory capacity of APC’s ↓, T cell reactivity restored 6–9 weeks after trt
Xueqing Wang 2020	120	Ib2–IIb	Surgery and NACT: TP or TC or TN	IgA, IgM, neutrophils, CD4, CD8	After trt	CD4/CD8 and NLR ratios ↑
Rui Li 2021	55	IIa–IVa	EBRT 50 Gy + cisplatin + HDR brachytherapy 25–36 Gy	CD3, CD4, CD8, PD-L1, PD-1, FoxP3CD25CD3, TCR	Before, midway (week 3), and after (week 8)	PD-1 on CD4 T cells ↓, CD4 and CD8 T cells ↓, FoxP3CD25CD3CD4 ↑, TCR diversity ↑

EBRT = external beam radiotherapy, BT = brachytherapy, CT = chemotherapy, HPT = hyperthermia, NACT = neoadjuvant chemotherapy, TP = cisplatin + paclitaxel, TC = carboplatin + paclitaxel, TN = nedaplatin, trt = treatment, PD = progressive disease, Th = T helper cells, Treg = regulatory T cells, NK cells = natural killer cells, NLR = neutrophil to lymphocyte ratio, SD = stable disease, PD = progressive disease, ↑ = increase, ↓ = decrease.

**Table 2:** Effects of (chemo) radiation on immune cells in TDLNs.

Author	N of pts	FIGO Stage	Therapy	Markers	Time Point	Results
Fattorossi 2004	38	Ia–IIIb	*n* = 15 surgery *n* = 4 CT (P) *n* = 19 CRT	NK’s, CD3, CD4, CD8,	At surgery PB not specified	After CRT: CD8 and NK cells ↑, activated CD4 ↑, activated CD8 ↑ compared to non trt pts, IFN-y producing CD4 and CD8 cells ↑ after CRT, Tregs unaffected by trt in TDLNs, Δ in TDLNs partly mirrored by Δ in PB
Battaglia 2007	14	Ib–IIa	*n* = 9 CRT (P based) *n* = 5 surgery	Nrp1+Tregs, Nrp1−Tregs	At surgery PB not specified	In TDLNs Nrp1+Tregs: FoxP3 and markers of activation ↑, Nrp1+Tregs: suppression of T cell proliferation in vitro, Nrp1+Tregs in PB did not display the same phenotypic features
Battaglia 2010	39	Ib–IVa	*n* = 11 CT (P) *n* = 9 CRT 40 Gy *n* = 19 CRT 50 Gy	NK’s, CD4, CD8, DC’s	At surgery PB pre trt, 5–6 weeks post trt	LD-TDLNs: Th1 and Tc1 ↑, and Tregs ↓, HD-TDLNs: Inverted CD4/CD8 ratio, Nrp1+Tregs ↑, CCR4+ Tregs ↑, unfavorable DC ratio

TDLNs = tumor draining lymph nodes, PB = peripheral blood, CRT = chemoradiation therapy, CT = chemotherapy, P = cisplatin, NK cells = natural killer cells, T regs = regulatory T cells, Th = T helper cells, Tc = cytotoxic T cells, trt = treatment, LD-TDLNs = low-dose-tumor draining lymph nodes, HD-TDLNs = high dose tumor draining lymph nodes, Δ = changes, ↑ = increase, ↓ = decrease.

**Table 3:** Effects of (chemo)radiation on tumor cells and TME.

Author	N of pts	FIGO Stage	Therapy	Markers	Time Point	Results
Qinfeng 2013	59	IIa–IIIb	RT (not specified) *n* = 59; 0 Gy *n* = 57; 0, 10 Gy *n* = 15; 0,10,20 Gy *n* = 8; 0,10,20,30 Gy	CD8, CD4, FoxP3, OX40	Before start, after 10, 20, 30 Gy resp	CD8 and CD4 more present in stroma, OX40 and FoxP3 similar in stroma and tumor before trt, CD8 and CD4 ↓ in stroma and tumor after trt, OX40 and FoxP3 = to pre-trt, stromal OX40 ↑ after 30 Gy
Dorta 2018	20	IbI–IIIb	CRT (platinum)	CD3, CD4, CD8, PD-1, FoxP3, CTLA-4	Before trt, after week 1,3,5	CD3, CD4, CD8, CD4FoxP3 ↓ after 1st week of trt, ↑ at week 3 and 5. Ki67, CD69CD8 ↑ in PB at week 3 and 5. No difference in CTLA-4, PD-1 PB and tumor. CTLA-4 and PD-1 on CD4 and CD8 stable
Berenguer Frances 2019	19	Ib–IIIb	EBRT(40 Gy)+CT(P)+ HDR brachy *n* = 7, PDR brachy *n* = 12	CD68, CD163, PD-L1	Before CRT, After CRT, 2 weeks after completion of BT	CD68 and CD163 = between HDR and PDR, CD68 and CD163 ↓ after HDR BT. PD-L1 ↑ 2 wks after HDR BT
Tsuchiya 2020	104	I–IV	EBRT(40 Gy)+CP *n* = 58, EBRT alone *n* = 46, Radical surgery *n* = 104	PD-L1, PD-1, HLA-1, CD8, FoxP3	Before start trt, After surgery	PD-L1 TCs ↑ after CRT, CD8 and FoxP3 infiltration ↓ after CRT, HLA-1 and PD-1 stable on ICs. CD8 and FoxP3 cells ↑ before trt predicts OS, PD-L1 ↑ in TCs predictive of OS and out of field recurrence
Cosper 2020	N = 20 (20/115)	Ib1–IIIb	EBRT 50.4 Gy +CP+BT	CD4, CD8 analysis, RNA seq	Before start trt, After three weeks CRT	In DOD pts: lymphocytes ↓ after 3 weeks, CD3e, CD4, CD8a, PRF1, GZMA and GNLY expression ↓, PD-L1, PD-L2 expression ↑, In NED pts: HPV gene expression ↓
Lippens 2020	38	Ib1–IVb	CRT+ CT (P)+ radical surgery	CD3, CD4, CD8, FoxP3, CD68, CD163, CD20, Il33, PD-L1	Before start trt Surgical specimen	pCR ↑: pre trt CD8 = CD3, CD8≥CD4, IL33+ tumor. DSS ↑: pre trt CD8/CD4 ↑, CD163/CD68 ratio ↑, PD-L1 >5%. pCR ↑: post trt CD8 ↓, CD163↓. Post trt CD3 ↑ and PD-L1 ↑: more metastases
Rui Lui 2021	55	IIa–IVa	EBRT 50 Gy +CT(P)+HDR BT 25–36 Gy	CD4, PD-L1, CD8, RNA sequencing	Before trt, mid-trt (3 weeks), and after trt (8 weeks)	PD-L1, CD4 and CD8 ↓ mid-trt and after trt, TCR diversity ↑ after trt

TME = tumor microenvironment, PB = peripheral blood, RT = radiotherapy, CRT = chemoradiation therapy, CT = chemotherapy, P = cisplatin, EBRT = external beam radiation therapy, BT = brachytherapy, HDR = high dose rate, PDR = pulsed dose rate, DOD = dead of disease, pCR = pathological complete response, NED = no evidence of disease, OS = overall survival, PFS = progression free survival, trt = treatment, TCR = T cell reactivity, ↑ = increase, ↓ = decrease.

**Table 4:** Pre-treatment immune state of the TME and its relation to treatment outcomes.

Author	N of pts	FIGO Stage	Therapy	Markers	Time Point	Results
Enwere 2017	120	Ib–IVa	CRT EBRT 45 Gy (P)	CD8, PD-L1	before trt	PD-L1 no relation to PFS or OS, Worse PFS in PD-L1 ↑and CD8 ↓ intratumoral
Rocha Martins 2019	21	IIb–IIIb	CRT (not specified)	CD68, CD8, PD-1, PD-L1, PD-L2	before trt	Stromal and intratumoral CD8 ↑, PD-1, PD-L1, PD-L2 ↑ in reponders
Someya 2020	81	IIa–IVb	EBRT 30–50 Gy BT CRT *n* = 63 Only RT *n* = 18	PD-L1, HLA-1, CD8, FoxP3	before trt	DSS ↑ in HLA-1+, PD-L1 no relation to PFS or OS, DSS ↑ if CD8, FoxP3 ↑
Someya 2021	100	IIa–IVb	EBRT 30–50 Gy ICBT 12–24 Gy CRT *n* = 77 RT *n* = 23	PD-L1, HLA-1, CD8, FoxP3,	before trt: inflamed, excluded, and cold type	Better prognosis when: FoxP3 ↑, inflamed and excluded tumor types
Komdeur 2017	460		RT/CRT + surgery *n* = 337 surgery alone *n* = 123	CD8CD103	before trt TCGA after trt	CD8CD103 ↑ and PFS ↑ in surgery alone
Kol 2021	501	Ia2–IVa	Surgery alone *n* = 251 or RT/CRT *n* = 255	STING CD103	before trt TCGA after trt	STING and CD103 ↑ associated with DSS ↑ and DFS ↑ in RT/CRT group

EBRT = external beam radiation therapy, ICBT = intra-cavitary brachytherapy, CRT = chemoradiation therapy, RT = radiotherapy, trt = treatment, PFS = progression free survival, OS = overall survival, DSS = disease specific survival, ↑ = increase, ↓ = decrease.

**Table 5:** TILs before and after NACT in the TME.

Author	N of pts	FIGO Stage	Therapy	Markers	Methods	Results
Meng 2018	97	I–IV	NACT *n* = 40	PD-L1, PD-1, CD8, HPV expression	IHC	PD-L1, PD-1, CD8 ↑ in advanced tumor and LVSI+, PD-L1, PD-1, CD8 ↑ after NACT
Liang 2018	137	Ib2–IIa	NACT: TP *n* = 105 BVP *n* = 22	CD8, FoxP3	IHC Intratumoral and peritumoral	intra- and peritumoral FoxP3 ↓ after NACT, CD8 = before vs. after NACT, pre-trt CD8 = in pCR vs. non pCR pts, FoxP3 ↓ in pCR, intratumoral CD8/peritumoral FoxP3 ↑ independent PFS and OS prognostic factor
Heeren 2019	13	Ib–IIb	NACT: TP *n* = 6 CP *n* = 7	CD3, CD8, FoxP3, Ki67, Tbet	IHC	After NACT (TP): Ki67+CD3+CD8- T cells ↓, FoxP3 ↓ in stroma CD8 ↑, TbetCD8 ↑ After NACT (P only): no changes in TILs
Liang 2020	142	Ib2–IIa	NACT: TP	PD-L1, CD3, CD4, CD8	IHC	After NACT: PD-L1 ↑, CD3, CD4, CD8 TILs no change and no relation with response, After NACT: CD8 associated with PD-L1, in responders PD-L1 ↓, when longer DFS PD-L1 ↓
Zhang 2020	109 N = 43 matched pre-post NACT	Ia–IVa	NACT: P based	CD3, CD4, CD8, CD56, CD68, PD-1, PD-L1	IHC, Scoring: immuno-active; immuno-medial, immuno-NK, immuno-deficient	CD4, CD8, CD20, CD56, PD-1 ↑ after trt, Ki-67 and PD-L1 ↓, CD4, CD8, CD20, CD56, PD-1 ↑ after trt in good responders, CD14 ↓ after NACT
Palaia 2021	37 N = 29 radical surgery	Ib2–IV	NACT: TP	Stromal TILs (not specified) PD-L1	IHC,	before trt: stromal TILs ↑ correlate with good response after NACT, after trt: TIL ↑ correlate with TC PD-L1 ↑, stromal TILs ↑ and NLR ↓ and PLR ↓ associated with better response
D’Alessandris 2021	38	Ib1–IV	NACT: TP	Stromal TILs: CD3, CD4, CD8, CD20, CD68, NCAM (NK) PD-L1	IHC	stroma: CD3/CD4, ↑ tumor: PD-L1 ↑ positively associated with TIL ↑, Correlation between % of TILs and PD-L1 expression on inlammatory cells

NLR = neutrophil-to-lymphocyte ratio, PLR = platelet-to-lymphocyte ratio, ELR = eosinophil-to-lymphocyte ratio, ENLR = eosinophil-to-lymphocyte ratio, TP = cisplatin + paclitaxel, P = paclitaxel, CP = cisplatin, BVP = vinca alkaloid and cisplatin, TIL = tumor infiltrating lymphocytes, TC = tumor cell, ↑ = increase, ↓ = decrease.
